# Physical Stability and Dissolution of Lumefantrine Amorphous Solid Dispersions Produced by Spray Anti-Solvent Precipitation

**DOI:** 10.1016/j.xphs.2020.12.033

**Published:** 2021-06

**Authors:** Sonal V. Bhujbal, Vaibhav Pathak, Dmitry Y. Zemlyanov, Lynne S. Taylor, Qi (Tony) Zhou

**Affiliations:** a Department of Industrial and Physical Pharmacy, College of Pharmacy, Purdue University, 575 Stadium Mall Drive, West Lafayette, IN 47907, USA; b Birck Nanotechnology Center, Purdue University, 1205 West State Street, West Lafayette, IN 47907, USA

**Keywords:** Amorphous solid dispersion, Precipitation, Moisture sorption, Physical stability, Dissolution

## Abstract

This study aims to develop amorphous solid dispersion (ASD) of lumefantrine with a cost-effective approach of spray anti-solvent precipitation. Four acidic polymers, hydroxypropylmethylcellulose phthalate (HPMCP), hydroxypropylmethylcellulose acetate succinate (HPMCAS), poly(methacrylic acid–ethyl acrylate) (EL100) and cellulose acetate phthalate (CAP) were studied as excipients at various drug-polymer ratios. Of the studied polymers, satisfactory physical stability was demonstrated for HPMCP- and HPMCAS-based ASDs with no observed powder X-ray diffraction peaks for up to 3 months of storage at 40 °C/75% RH. HPMCP and HPMCAS ASDs also achieved greater drug release levels in the dissolution study than other polymers. The HPMCP-based ASDs with a drug:polymer ratio of 2:8 exhibited a maximum drug release of 140 μg/mL for up to 2 h, which is significantly higher than the currently marketed formulation of Coartem® (<80 ng/mL). Relatively, the CAP and EL100 ASDs indicated a higher water content and crystallized within a day when stored at 40 °C/75% RH. The choice of polymer, and the drug-polymer ratio played a crucial role in the solubility enhancement of lumefantrine. Our study indicates that the developed spray anti-solvent precipitation method could be an affordable approach for producing ASDs.

## Introduction

Due to an increase in the number of therapeutic compounds characterized by poor aqueous solubility, formulation strategies for enhancing drug solubility are in demand. Some commonly-used strategies are amorphization, complexation, salt formation, and micellization.^1^^,^^2^ Drug amorphization is popular because it not only enhances the drug solubility and dissolution rate but also results in the generation of a supersaturated solution which creates higher flux across the intestinal membrane.^3^ However, the amorphous state is thermodynamically unstable and may revert to the crystalline form. If this conversion is too rapid, the amorphous drug can lose its dissolution advantage, causing quality and efficacy issues. To inhibit crystallization, an amorphous drug is often formulated with a polymer to form an amorphous solid dispersion (ASD).^4^^,^^5^ Different mechanisms have been proposed regarding how the polymers inhibit crystallization of amorphous drugs, including anti-plasticization,^6^^,^^7^ interactions between drug and polymer in a dispersion,^8^, ^9^, ^10^ reduced molecular mobility,^11^ and an increased energy barrier for crystal nucleation.^12^^,^^13^

Various processes such as anti-solvent precipitation,^14^ freeze-drying,^15^ milling,^16^ solvent impregnation,^17^ etc have been applied to manufacture ASDs. The most common industrial manufacturing approaches for ASDs are spray drying and hot-melt extrusion (HME).^18^^,^^19^ However, spray drying and HME have disadvantages. Spray drying utilizes a large amount of organic solvents, which can lead to challenges controlling the residual solvent content. The final spray-dried product often has low bulk density and high electrostatic charge.^20^ The use of HME requires the components to be stable at high processing temperatures, making it difficult to process thermolabile compounds. In addition, spray drying and melt extrusion have a relatively high capital cost of equipment and often require skilled personnel. In addition, the usage of a large volume of organic solvent, maintenance of inert atmosphere, and collection of the organic solvent for spray-dried formulations further add on to associated costs. Maintenance of high temperatures makes the HME a method relatively expensive which inhibits their application in under-developed and developing countries. The goal of this work was to develop a preparation method for ASD which is associated with lower costs. Therefore, an alternative method of anti-solvent precipitation was explored in this study.

Antisolvent co-precipitation was one of the first routes explored to evaluate amorphous dispersions.^21^^,^^22^ This method has been successfully used to formulate ASDs of poorly soluble drugs on a small scale^23^, ^24^, ^25^, ^26^, ^27^ as well as industrial-scale.^28^^,^^29^ In this method, a drug-polymer solution (usually in a non-volatile solvent) is added to its anti-solvent (usually a cold aqueous solvent), leading to the precipitation of ASDs. These ASDs are then washed and dried. The drug-polymer system should be insoluble in the anti-solvent, and the anti-solvent should be miscible with the organic solvent used to dissolve drug and polymer. This method is particularly advantageous for compounds exhibiting both low solubility in volatile solvents and high melting points with a propensity for degradation (of either the drug or polymer) at elevated temperatures.^14^ As described, anti-solvent precipitation is a simple method with a relatively cheaper manufacturing set-up than spray drying or HME. This method requires less organic solvent (relative to spray drying), and does not require maintenance of inert ambiance due to the non-volatile nature of the solvent. Therefore, even considering associated costs for maintaining cold anti-solvent, washing/filtration to remove solvent residue, and recovering ASDs, the anti-solvent precipitation method is a cost-effective alternative to spray drying and HME. Most importantly, since it requires a simple set-up with low capital investment, it has a strong potential for a wider application in developing countries.

However, anti-solvent precipitation poses its own challenges. Firstly, it is crucial to determine the optimal solvent-*anti*-solvent combination for the drug-polymer system of interest to obtain ASD precipitation. Secondly, relative to other methods, anti-solvent precipitation involves the use of an aqueous solvent to a much higher extent and the ASDs are in contact with water for a longer duration. Such higher exposure to moisture, which is a known factor accelerating the crystallization of amorphous drugs^30^^,^^31^ may cause physical instability in ASDs by recrystallizing the drug. Hence, it is essential to ensure that the aqueous solvent does not compromise the drug amorphization process and that the residual moisture is removed rapidly. In this study, the feasibility of preparing ASDs by an anti-solvent precipitation method was studied for lumefantrine.

Lumefantrine is an anti-malarial drug used in combination with artemether (artemisinin derivative). Lumefantrine has poor absorption due to its low aqueous solubility (<80 ng/mL). The low bioavailability of 4–11% often leads to high drug dosage, higher treatment cost, and subsequently poorer patient compliance.^32^ Due to the poor absorption of lumefantrine, there have been reported cases of treatment failure.^33^^,^^34^ Therefore, it is crucial to develop lumefantrine products with improved solubility and bioavailability. Since malaria is prevalent in low-income countries, the low-cost anti-solvent precipitation approach could be an affordable choice to prepare ASDs of lumefantrine. The objectives of this study were to (1) develop ASDs of lumefantrine by the anti-solvent precipitation method; (2) characterize the ASDs regarding their physicochemical properties, drug release profile, and solid-state stability; and (3) determine the optimal polymer choice and drug-polymer composition for manufacturing ASDs of lumefantrine using anti-solvent precipitation.

## Materials

**Fig. 1:**
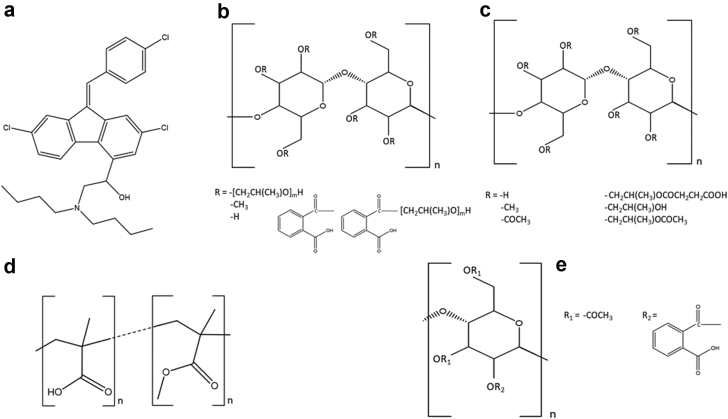
Chemical structures of (a) lumefantrine, (b) HPMCP, (c) HPMCAS, (d) EL100, and (e) CAP.

Lumefantrine was supplied by Euroasias (Mumbai, India). Polymers used in the study were obtained from different sources: poly(methacrylic acid–ethyl acrylate) (Eudragit L100) from Degussa Rohm Pharma Polymers (Darmstadt, Germany), cellulose acetate phthalate from Sigma-Aldrich (St. Louis, MO), hypromellose phthalate (HPMCP-50) and hypromellose acetate succinate (HPMCAS-MF) from Shin-Etsu Chemicals (Tokyo, Japan). The solvent dimethylacetamide (DMA) was purchased from Sigma-Aldrich (St. Louis, MO). The chemical structure of lumefantrine and polymers are shown in Fig. 1.

## Methods

### Preparation of ASDs

Lumefantrine (lum) ASDs were prepared using spray anti-solvent precipitation with four acidic polymers: hypromellose phthalate (HPMCP), hypromellose acetate succinate (HPMCAS), cellulose acetate phthalate (CAP), and Eudragit L100 (EL100). For a 300 mg batch, drug and polymer (in 4:6 ratio) were dissolved in 5 mL dimethylacetamide (DMA) and the solution was sprayed using an ultrasonic nozzle (Büchi Labortechnik AG, Falwil, Switzerland) into 200 mL ice-cold acidified water (0.02 N HCl) while stirring. The resulting aqueous suspension was vacuum-filtered to collect the solid particles. The solids were placed in an open Petri dish and dried in a Scienceware® vacuum desiccator from Sigma-Aldrich (St. Louis, MO) with at room temperature. The duration of drying varied between 12 and 100 h depending upon the sample. The drug-polymer combinations exhibiting satisfactory stability and dissolution properties were investigated further with additional drug-polymer ratios of 2:8 and 3:7. Pure amorphous lumefantrine, for the purpose of analysis, was prepared by melt-quench method. For this, 50 mg of lumefantrine was melted in an aluminum pan on a hot plate at 150 °C and was then immersed in liquid nitrogen. It was then powdered by grinding with mortar and pestle. The amorphous state was confirmed by PXRD analysis (supplementary data).

### Powder X-ray Diffraction (PXRD)

The X-ray diffraction profile of the powders was determined using the Rigaku Smartlab™ diffractometer (Rigaku Americas, The Woodlands, TX, USA). The instrument has a Cu-Kα radiation source and a D/tex ultra detector. The samples were mounted on a glass sample holder and staged on the instrument. The radiation source was operated at 40 kV voltage and 44 mA current. The X-ray diffraction patterns were obtained in a 2θ range of 4–40° at a scan speed of 4°/min and a resolution of 0.02°.

### Fourier-Transform Infrared Spectroscopy (FTIR)

FTIR spectra of powder samples were obtained using a Vertex 70 IR Spectrophotometer (Bruker Optics, Billerica, MA, USA) with a golden gate attenuated total reflectance (ATR) accessory (Specac, Fort Washington, PA, USA). Dry air was purged through the ATR cell and the detector unit during the analysis. A background scan was recorded before each sample measurement and the spectra were obtained in the range of 400–4000 cm^−1^. Each spectrum was obtained as an average of 64 scans.

### X-ray Photoelectron Spectroscopy (XPS)

XPS (AXIS Ultra DLD spectrometer, Kratos Analytical Inc., Manchester, UK) analysis was performed to determine the surface composition of ASD solids. Monochromatic Al Kα radiation (1486.6 eV) was utilized for high-resolution (with a pass energy of 20 eV) and survey spectra (with a pass energy of 160 eV). In order to avoid non-homogenous electric charging of powder sample and to obtain higher resolution, a commercial Kratos charge neutralizer was employed. The instrument resolution was approximately 0.35 eV at pass energy of 20 eV. Calibration of binding energy (BE) values was performed using Au 4f_7/2_ at 84.0 eV and Cu 2p_3/2_ at 932.67 eV. Powder samples were attached to a stainless-steel sample holder using copper tape.

XPS data analysis was conducted using CasaXPS software (version 2313 Dev64). First, the *C*–C component of the C 1s peak was fixed to 284.6 eV BE for charge correction. Thereafter, from the reference peaks of pure compounds, a Shirley background correction was made. Finally, the CasaXPS software was used to factor in the Scofield atomic sensitivity and inelastic mean free path of photoelectrons to estimate the atomic concentration of elements on the surface, i.e. approximately 10 nm.

### Differential Scanning Calorimetry (DSC)

The thermal characteristics of ASDs and pure materials were determined using a modulated DSC (Q2000 model, TA Instruments, New Castle, DE, USA). The samples were purged with dry nitrogen gas at 50 mL/min during analysis. The instrument was calibrated using indium and tin. About 3–5 mg of sample, was placed in a non-hermetically sealed aluminum Tzero pan (TA Instruments, New Castle, DE, USA), was first cooled to −25 °C and then heated to 200 °C at a heating rate of 5 °C/min and modulation of 2 °C every 60 s. The Universal Analysis 2000 software was used to determine the glass transition temperature (T_g_) by analyzing reversible heat flow.

### Thermogravimetry (TGA)

The weight change of ASD powder samples as a function of temperature was determined using a PerkinElmer TGA 4000 thermogravimetric analyzer (Waltham, MA, USA). About 4–6 mg of sample was placed in a ceramic sample cup and heated up to 170 °C at a rate of 10 °C/min. The sample chamber was continuously purged with nitrogen gas at a flow rate of 20 mL/min during the analysis. Triplicate measurements were conducted for each batch and results were averaged. The ASD water content was estimated by calculating the percent loss in weight between 35 °C and 170 °C.

### Scanning Electron Microscopy

The particle morphology of ASD powders was examined using a NOVA nanoSEM, a field emission scanning electron microscope (FEI Company, Hillsboro, OR, USA). A small amount of powder sample was attached to a stainless-steel sample holder using double-sided carbon tape. Excess particles were removed using pressurized air. The attached sample was then coated with platinum using a sputter coater for 60 s (208 HR, Cressington Sputter Coater, Watford, WD, UK). The sample holder with the coated sample was then placed in the SEM instrument. The instrument was operated using the accompanying software and images of the powder samples were captured at various magnifications.

### Particle Size Distribution

The particle size distribution of powders was determined using a Malvern Mastersizer 3000, a laser diffraction-based instrument (Malvern, UK). HYDRO LV - a liquid-based dispersion accessory - was used to disperse the powder sample for analysis. Because the lumefantrine ASDs are insoluble in acidified water, we used acidified water (0.02 N HCl, pH 1.7) as the powder dispersant. The instrument initialization, laser alignment, and background measurement were executed prior to each sample measurement using the instrument software. To start the measurement, each sample was manually added into the dispersant under constant stirring and circulation in the diffraction cell. The sample was added until the laser obscuration value was reached and stabilized within a range of 5–20% prior to executing the sample measurement. For each sample, the size distribution was measured in triplicate and average distribution data were generated. The average percentile sizes d_10_, d_50_, and d_90_ were obtained for each sample.

### Dissolution

The dissolution study of lumefantrine ASDs was performed in 100 mL of 50 mM pH 6.8 phosphate buffer contained in a jacketed 100 mL beaker. The dissolution media was maintained at 37 °C and stirred at 300 rpm. The ASD powders containing 20 mg drug were added into the dissolution media. Non-sink conditions were maintained to evaluate the extent of supersaturation generation.^35^^,^^36^ During dissolution, about 1 mL of the solution was withdrawn and replaced with 1 mL of the buffer at various time points up to 2 h. The withdrawn samples were filtered using a 0.22-μm nylon syringe filter before HPLC analysis. The dissolution profile was calculated by taking the mean of triplicate samples.

HPLC analysis of lumefantrine was performed using an Agilent HPLC 1260 Infinity II system (Agilent Technologies, Santa Clara, CA) equipped with a diode array detector. The method is modified from the previous reports.^37^ An Agilent Eclipse Plus C18 column (5 μm, 150 × 4.6 mm, Agilent, Waldbronn, Germany) was used at 25 °C. The mobile phase was a 75:25 v/v mixture of acetonitrile and 30 mM pH 2.5 sodium sulfate buffer with an isocratic flow of 1 mL/min. The wavelength used for lumefantrine detection was 380 nm and the retention time for lumefantrine was 3.9 min. A calibration curve was prepared for lumefantrine (R^2^ > 0.999) with concentrations ranging from 1 to 500 μg/mL.

### Statistical Analysis

One-way analysis of variance (ANOVA) and Tukey's honestly significant difference (HSD) tests (α = 5%) were used to determine the statistical difference between different samples. The statistical analysis was conducted using SAS 9.4 software (SAS Institute Inc., Cary, NC, USA). The data are presented as mean ± standard deviation.

## Results

### Dispersion Crystallinity

**Fig. 2:**
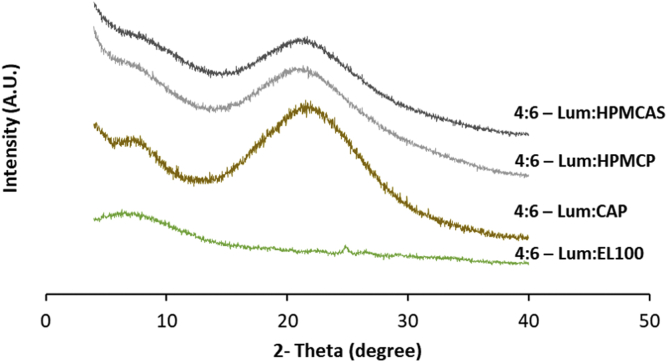
X-ray diffraction patterns of lumefantrine dispersions with various polymers at a drug:polymer ratio of 4:6 as prepared by anti-solvent precipitation immediately after preparation.

**Fig. 3:**
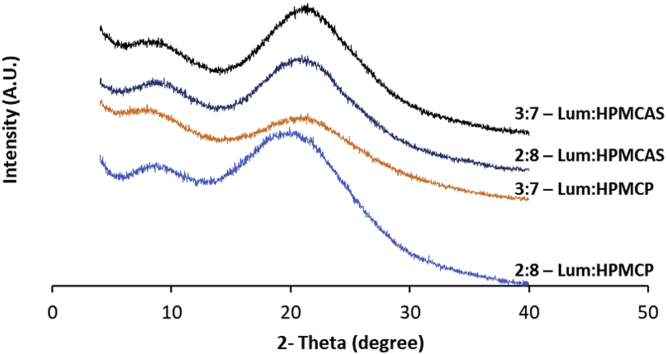
X-ray diffraction patterns of lumefantrine dispersions with HPMCP and HPMCAS at a drug:polymer ratio of 2:8 and 3:7 as prepared by antisolvent precipitation immediately following preparation.

The PXRD diffractograms of ASDs obtained immediately following the drying indicated no crystallinity, based on an absence of diffraction peaks, at drug-polymer ratios of 4:6 for HPMCP, HPMCAS, and CAP; while ASDs with EL100 showed small peaks (Fig. 2) consistent with crystalline lumefantrine (Fig. S1). Even lower drug-polymer ratios with EL100 (2:8 and 3:7) showed diffraction peaks consistent with the crystallization of lumefantrine immediately upon preparation (data not shown). Since preliminary results showed that the HPMCP- and HPMCAS-based ASDs had better stability over other polymers, additional drug-polymer ratios of 2:8 and 3:7 were prepared and further investigated for these two polymers. ASDs with drug-polymer ratios of 2:8 and 3:7 for HPMCP and HPMCAS did not show diffractions peaks after preparation (Fig. 3). The physical stability of ASDs prepared with HPMCP, HPMCAS, and CAP was further studied at the accelerated storage condition of 40 °C/75% RH for three months.

**Fig. 4:**
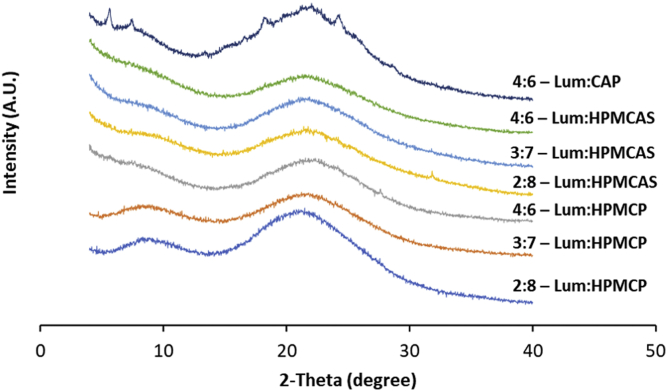
X-ray diffraction patterns of selected dispersions after storage at 40 °C/75% RH for up to 3 months.

The ASD with lumefantrine:CAP at a ratio of 4:6 began to crystallize within the first day of storage as shown in Fig. 4. However, there was no increase in the diffraction peak areas over a period of 3 months under accelerated storage conditions. Also, the dispersion did not develop crystallinity over three months when stored under a vacuum. The 2:8, 3:7, and 4:6 lumefantrine:HPMCP dispersions showed no crystallinity over 3 months of storage, indicating prolonged retention of the amorphous state. Similarly, no peaks were detected for the 2:8, 3:7, and 4:6 HPMCAS-based dispersions for 3 months. The PXRD diffractograms of all of the HPMCP and HPMCAS-based dispersions after 3 months of storage at 40 °C/75% RH are shown in Fig. 4. In summary, HPMCP and HPMCAS-based solid dispersions were found to be relatively stable.

### Fourier Transform-Infrared Spectroscopy (FTIR)

**Fig. 5:**
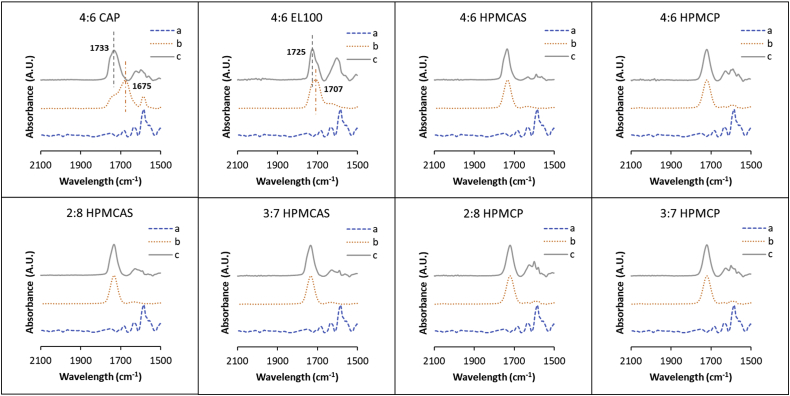
FTIR Spectra of carbonyl regions (1500–2100 cm^−1^) of (a) amorphous lumefantrine, (b) pure polymer, and (c) ASDs.

A carbonyl peak of the polymer shifted to higher wavenumbers after the formation of dispersion with lumefantrine (Fig. 5). However, the extent of the shift varied for different polymers. The most remarkable shift was in the 4:6 lum:CAP ASD, which showed a dominant carbonyl peak at 1733 cm^−1^ in the dispersion, relative to the main carbonyl peak at 1675 cm^−1^ in the neat CAP polymer. A similar shift in the main carbonyl peak was observed in the EL100 dispersion from 1707 to 1725 cm^−1^. However, only minor changes were observed for HPMCP and HPMCAS dispersions. The spectra of dispersions with 20% and 30% drug loading for HPMCAS and HPMCP were similar to that of a corresponding 40% drug loading. It should be noted that all of the polymers contain carbonyl groups arising from two functional groups; an ester or an acid group. The spectrum of neat CAP clearly shows that the main carbonyl peak has a notable shoulder (at a higher wavenumber), consistent with two chemically distinct carbonyl groups. Further, the change in the peak ratio in the lumefantrine dispersion further suggests an interaction of the polymer with the drug that changes the chemical environment of one of the carbonyl groups. For other polymers, it is not possible to resolve two carbonyl peaks in the neat polymers, although subtle differences to peak shape in the presence of lumefantrine again suggest changes in the chemical environment of the carbonyl groups. Given the acidic functional groups in the polymer, the basic nature of lumefantrine, and the changes in the IR spectrum, additional probing of the exact nature of the drug-polymer interaction is warranted. Consequently, XPS analysis was performed to evaluate the ionization state of lumefantrine.

### X-ray Photoelectron Spectroscopy (XPS)

Samples were analyzed using XPS in order to further evaluate the interaction of polymer and lumefantrine. As observed previously, neat lumefantrine shows a single N1s peak with a binding energy at 399 eV. However, if the nitrogen is protonated due to the presence of the acidic polymers, a second N1s peak emerges with a binding energy of 402 eV.^38^^,^^39^ This higher binding energy is consistent with the greater energy required to remove electrons in the presence of a positive charge. Peak fitting was performed on the XPS data to calculate the percentage of protonation. Table 1 shows the extent of protonation of the tertiary nitrogen of lumefantrine in the various ASDs. A significant protonation of the nitrogen by polymers can be seen for the ASDs, relative to the amorphous lumefantrine alone. The most protonation was seen in the HPMCP and EL100 systems, followed by CAP. HPMCAS was the least protonated amongst the ASDs. There also appears to be some correlation between the extent of polymer loading and lumefantrine protonation, where the extent of protonation increases with an increase in polymer loading.

**Table 1:** Average Percent Protonation of Lumefantrine in ASDs (n = 3).

Sample	% Protonation
2:8 Lum:HPMCP	42 ± 3
3:7 Lum:HPMCP	40 ± 2
4:6 Lum:HPMCP	37 ± 3
2:8 Lum:HPMCAS	37 ± 4
3:7 Lum:HPMCAS	35 ± 7
4:6 Lum:HPMCAS	14 ± 2
4:6 Lum:CAP	32 ± 2
4:6 Lum:EL100	38 ± 4
Amorphous lumefantrine	3 ± 1

### Thermal Analysis

The glass transition temperature (T_g_) and the water content of various dispersions are summarized in Table 2 and Fig. S2. Except for EL100 ASDs, no melting peak was observed for any of the dispersions, indicating that the drug was amorphous at all the studied drug loadings for HPMCP, HPMCAS, and CAP. At the 4:6 drug-polymer ratio, the T_g_ values of HPMCP and HPMCAS ASDs were between the T_g_ of neat amorphous drug and neat polymer. The T_g_ values of CAP ASD were similar to those of HPMCAS ASDs, even though neat CAP exhibits a much higher T_g_ than pure HPMCAS. This could be due to the higher water content of CAP ASDs. The average water content of the ASDs ranged from 4 to 6%, except for the 4:6 CAP dispersion, which had approximately 10%. The higher water content in the CAP dispersion could also be responsible for rapid crystallization under the accelerated storage conditions.

**Table 2:** Glass Transition Temperature (T_g_) and Moisture Content of Lumefantrine Dispersions with T_g_ Values of Pure Polymer and Drug (n = 3).

Samples	T_g_ (°C)	2:8 Lum:Polymer		3:7 Lum:Polymer		4:6 Lum:Polymer	
		T_g_ (°C)	% Moisture Content	T_g_ (°C)	% Moisture Content	T_g_ (°C)	% Moisture Content
Lum	18	–	–	–	–	–	–
HPMCP	150	88 ± 2	5 ± 0	112 ± 19	5 ± 0	110 ± 5	4 ± 1
HPMCAS	117	45 ± 2	5 ± 0	101 ± 22	6 ± 0	87 ± 1	5 ± 0
CAP	133	Not performed				82 ± 4	9 ± 0

The EL100 based ASDs and lower drug loadings of CAP-based ASDs were not tested due to the poor stability profile exhibited in the earlier studies.

The ASDs with HPMCP showed higher T_g_ values than ASDs with different polymers at each drug loading. The thermal analysis results were in general agreement with the PXRD results, suggesting that HPMCP-based lumefantrine solid dispersions were more physically stable than HPMCAS and CAP dispersions. The EL100-based ASD appeared the least stable of the tested formulations and hence were excluded from further studies.

### Particle Morphology and Size Distribution

**Fig. 6:**
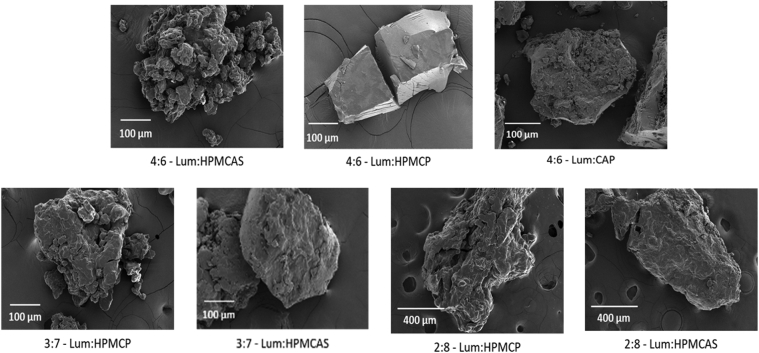
Representative SEM images of lumefantrine ASDs.

The representative SEM images of ASDs are shown in Fig. 6. The ASD particles were irregularly shaped and exhibited a tendency to aggregate, creating a larger particle size. Particle size is an important factor in the dissolution performance of ASDs. In order to measure the extent of particle size increase upon drying and to characterize the particle size for further dissolution studies, the particle size distribution of each ASD dispersion was measured at two stages of preparation: (i) immediately after anti-solvent precipitation (i.e. in the suspension form before drying), and (ii) after vacuum drying. The dried powder form was utilized for dissolution studies. The particle size distributions of all samples are summarized in Table 3. For all the HPMCAS and CAP ASDs, vacuum-dried particles showed significantly larger sizes than the corresponding suspension form. For 2:8 and 4:6 HPMCP ASDs, the vacuum-dried form showed higher values of d_10_ and d_50_ than the corresponding suspension form; however, the d_90_ values were similar for both forms. Interestingly, 3:7 HPMCP ASD showed higher percentile sizes in the suspension form than the vacuum-dried form.

**Table 3:** Particle Sizes and Span of ASDs Before and After Vacuum Drying (n = 3).

Polymer	Drug %	d_10_ (μm)	d_50_ (μm)	d_90_ (μm)	Span
HPMCP	20%	36.8 ± 1.6	139.0 ± 9.9	335.0 ± 33.5	2.15 ± 0.1
	30%	29.5 ± 0.3	99.2 ± 1.4	268.0 ± 5.7	2.40 ± 0.0
	40%	31.9 ± 0.6	116.0 ± 3.6	378 ± 29.5	2.98 ± 0.2
HPMCP Dried	20%	60.8 ± 0.4	160 ± 1.4	367 ± 11.8	1.91 ± 0.1
	30%	19.8 ± 0.3	71.5 ± 0.8	213 ± 11.0	2.7 ± 0.1
	40%	43.9 ± 2.2	147 ± 8.6	563 ± 161.3	3.53 ± 0.8
HPMCAS	20%	19.5 ± 0.2	62 ± 2.1	197.0 ± 24.1	2.86 ± 0.3
	30%	19.3 ± 0.7	56.2 ± 4.9	156.0 ± 22.8	2.43 ± 0.2
	40%	18.9 ± 0.1	65.0 ± 2.2	171.0 ± 7.0	2.34 ± 0.0
HPMCAS Dried	20%	68.7 ± 8.7	1180 ± 90.0	2505 ± 15.0	2.06 ± 0.2
	30%	172 ± 4.9	444 ± 37.7	1870 ± 335.1	3.82 ± 0.5
	40%	75.6 ± 1.7	366 ± 16.9	1130 ± 131.0	2.88 ± 0.4
CAP	40%	24 ± 1.1	76.7 ± 5.9	183 ± 20.2	2.07 ± 0.1
CAP Dried	40%	28.1 ± 0.3	141 ± 6.8	575 ± 40.4	3.88 ± 0.1

### Dissolution

**Fig. 7:**
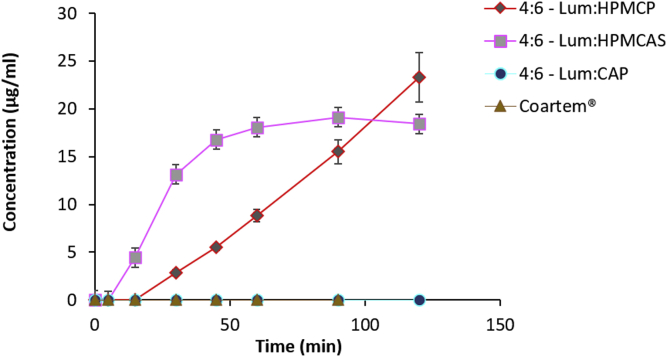
Drug dissolution profiles of Coartem® and 4:6 Lum:polymer dispersions with HPMCP, HPMCAS, and CAP in 100 mL of 50 mM pH 6.8 buffer (n = 3).

The drug dissolution profiles of 4:6 lum-polymer dispersions in 100 mL of 50 mM pH 6.8 phosphate buffer are shown in Fig. 7. The amount of lumefantrine drug released from HPMCP and HPMCAS ASDs after 30 min exceeded the drug's amorphous solubility of 2 μg/mL. In contrast, no drug release was detected for CAP ASD after 2 h. At a drug-polymer ratio of 4:6, HPMCAS ASD dissolved rapidly and reached a plateaued drug concentration after 1 h. The dissolution of 4:6 HPMCP ASD occurred steadily with a monotonic increase in drug concentration over time. After 2 h of dissolution, HPMCAS and HPMCP ASDs reached a drug concentration of 18.4 and 23.3 μg/mL respectively. These two ASDs showed a substantially higher drug release relative to Coartem®.

**Fig. 8:**
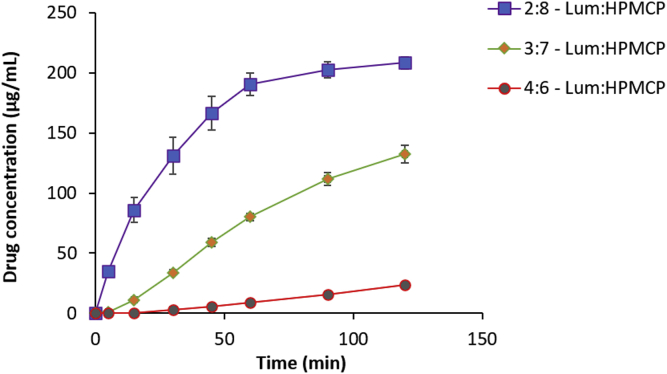
Drug dissolution profiles of 2:8, 3:7 and 4:6 Lum:HPMCP dispersions in 100 mL of 50 mM pH 6.8 buffer (n = 3).

**Fig. 9:**
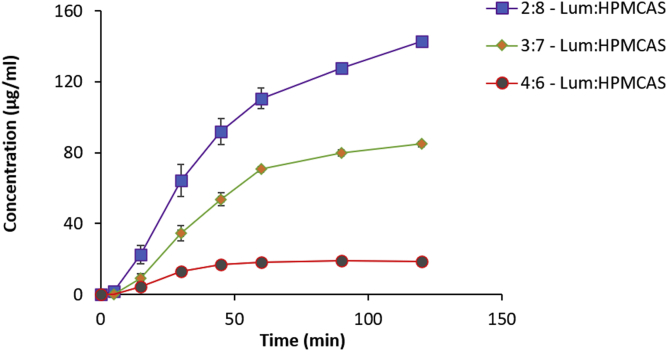
Drug dissolution profiles of 2:8, 3:7 and 4:6 Lum:HPMCAS dispersions in 100 mL of 50 mM pH 6.8 buffer (n = 3).

The dissolution performance was also determined at a drug/polymer loading of 2:8 and 3:7 for HPMCP and HPMCAS (Fig. 8, Fig. 9). The drug release increased with increasing polymer fraction in both instances. HPMCP provided a significantly higher drug release than HPMCAS for each drug-polymer ratio. At the 2:8 drug/polymer ratio, HPMCP had a final drug concentration of 208 μg/mL at 2 h while HPMCAS reached 143 μg/mL. Both ASDs showed rapid drug release in the first hour followed by a slower rate of release. At the end of 2 h, the 2:8 HPMCP ASD attained almost 100% drug release while the 2:8 HPMCAS ASD released 71% of the drug. At the 2:8 ratio, the HPMCP and HPMCAS-based ASDs released about 100- and 60-times more lumefantrine into the solution than the lumefantrine amorphous solubility (1.8 μg/mL).

The release performance at the 3:7 drug-polymer ratio shows significant improvement over the 4:6 ratio for both polymers. The 3:7 lum-HPMCP ASD released drug at a steady rate to reach a final concentration of 132.6 μg/mL. The 3:7 HPMCAS ASD dissolved rapidly in the first hour followed by a slower dissolution rate before attaining a final released concentration of 84.8 μg/mL. At this drug-polymer ratio, the percent drug release after 2 h was 66% for HPMCP and 42% for HPMCAS. The smaller particle sizes of HPMCP ASDs relative to the corresponding HPMCAS ASDs may explain their better dissolution.

### Powder Flow Property

Since amongst the tested stable formulations, 2:8 Lum:HPMCP dispersions indicated almost complete drug release, and 4:6 Lum:HPMCP dispersions exhibited maximum drug release at a high drug load of 40% w/w, they were further assessed for their powder flow properties. The flowability was measured using Carr's compressibility index (CI) and Hausner's ratio (HR). The CI and HR values for 2:8 Lum:HPMCP dispersions were 7.1 ± 1.2% and 1.1 ± 0.0% respectively, whereas CI and HR values for 4:6 Lum:HPMCP dispersions were 6.3 ± 3.7% and 1.1 ± 0.0% respectively (Table S1). These results indicate that this manufacturing approach leads to powders of excellent flow behavior and compressibility properties.

## Discussion

The study demonstrated that the choice of polymer critically affected the physical stability of lumefantrine ASDs prepared by anti-solvent precipitation. Over the duration of the stability study (3 months), HPMCP and HPMCAS dispersions showed no crystallization at the tested drug loadings. This is a surprising behavior considering the involvement of water as an anti-solvent. Significantly higher water content in the CAP dispersion after drying could be a reason for the rapid crystallization within a day of storage. EL100-based ASDs did not have a high moisture content after drying but these dispersions took much longer to dry (4 days) than others (~12 h). Such prolonged contact of the amorphous drug with water is known to enhance the risk for crystallization and instability.^46^ In order to overcome the challenges that aqueous solvent poses to ASDs, there have been studies in the past which used organic solvents only for anti-solvent precipitation process.^47^ However, this approach was not explored in this study because this could add to the potential costs of lumefantrine ASD products prepared using this method, and because stable lumefantrine ASDs with good dissolution performance were formulated with HPMCP and HPMCAS polymers even while using the aqueous solvents. In future studies, a more efficient drying system should be considered to reduce the ASD contact time with water.

The polymers have much higher Tg values than the drug in this study. It is interesting that Tg value of 2:8 Lum:polymer ASD is much lower than those of 3:7 Lum:polymer and 4:8 Lum:polymer. It seems the Tg values of our ASD systems are affected by a set of factors rather than just according to the ratio of drug and polymer. Theoretically, a miscible ASD system should have a Tg value that is between the Tg of the neat drug and neat polymer.^40^, ^41^, ^42^ The presence of water could decrease the Tg^43^; however, in our study moisture contents of different ASDs are similar, which unlikely contribute to the Tg difference significantly. Other factors including formation of ionization may also affect the Tg of the ASDs.^44^^,^^45^ Further studies are warranted to explore such surprising phenomenon.

Interestingly, in a previous study where ASDs were prepared using the solvent evaporation technique, where no aqueous solvents were involved, a strong correlation was found between the degree of protonation in lumefantrine and its physical stability. The ASDs having a higher extent of lumefantrine protonation exhibiting better stability.^39^ However, in this study at the 4:6 drug:polymer ratio, the extent of protonation in lumefantrine in the ASDs with different polymers was ranked HPMCP > EL100 > CAP > HPMCAS > amorphous lumefantrine, while the order of lumefantrine stability was HPMCP > HPMCAS > CAP > EL100 > amorphous lumefantrine. From these results, a correlation is not observed between the degree of protonation in lumefantrine and ASD stability. However, it is noteworthy that both CAP- and EL100-based systems, which both exhibited higher degrees of lumefantrine protonation, also had higher moisture contents or were exposed to moisture for a longer time due to slow drying rates. When no aqueous solvent was involved in the preparation process, EL100 and CAP based lumefantrine ASDs exhibited much higher stability (>30 days under accelerated storage conditions),^39^ than when the aqueous solvent was involved like in this study. A high moisture content or prolonged exposure to moisture could have been a cause of instability in these ASDs. On the other hand, the HPMCP- and HPMCAS-based ASD systems with a higher protonation than amorphous lumefantrine do exhibit higher stability. Thus, a higher degree of tertiary nitrogen protonation in lumefantrine may confer greater stability to the ASD while the presence of high moisture content negatively affects stability.

The HPMCP- and HPMCAS-based systems exhibited maximum drug release amongst the tested ASDs. At the 2:8 drug-polymer ratio, the HPMCP-based ASD showed a final drug concentration of 208.4 μg/mL while the corresponding HPMCAS-based ASD reached 142.8 μg/mL. These values are 100- and 60- times higher than the lumefantrine's amorphous solubility of 1.8 μg/mL. At a higher drug load of 3:7 drug-polymer ratio, HPMCP exhibited a drug release of 132.6 μg/mL. The relatively poorer drug release from the CAP-based ASDs was also seen in previous studies where CAP-based ASDs had a low lumefantrine release. Trasi et al. found that at 40% drug load, the lumefantrine-CAP system exhibited an incongruent release pattern, wherein the polymer released rapidly but the drug released very slowly. They also discovered that decreasing drug loads below 40% in the CAP-based ASDs achieved greater lumefantrine release.^39^ This poor drug release from the CAP-based ASDs could be related to the stronger drug-polymer interactions as seen from the high degree of protonation observed for this system. However, more studies are warranted to better understand this phenomenon.

## Conclusions

In this study, we have successfully prepared stable lumefantrine ASDs with substantially improved dissolution using a spray anti-solvent precipitation method. The results indicate that the choice of polymer and drug-polymer ratio played an important role in the physical stability and dissolution behavior of lumefantrine ASDs. The stability of the ASDs was reduced by the presence of moisture during manufacturing or storage. The ASD with the drug:HPMCP ratio of 2:8 showed the best stability and drug release. Our study demonstrated that spray anti-solvent precipitation is a viable and economical approach to manufacture amorphous solid dispersions with satisfactory physical stability and drug release.

## References

[1] Baghel S., Cathcart H., O'Reilly N.J. (2016). Polymeric amorphous solid dispersions: a review of amorphization, crystallization, stabilization, solid-state characterization, and aqueous solubilization of biopharmaceutical classification system class II drugs. J Pharm Sci.

[2] Vemula V.R., Lagishetty V., Lingala S. (2010). Solubility enhancement techniques. Int J Pharm Sci Rev Res.

[3] Frank K.J., Rosenblatt K.M., Westedt U. (2012). Amorphous solid dispersion enhances permeation of poorly soluble ABT-102: true supersaturation vs. apparent solubility enhancement. Int J Pharm.

[4] Chiou W.L., Riegelman S. (1971). Pharmaceutical applications of solid dispersion systems. J Pharm Sci.

[5] Serajuddin A.T. (1999). Solid dispersion of poorly water-soluble drugs: early promises, subsequent problems, and recent breakthroughs. J Pharm Sci.

[6] Van den Mooter G., Wuyts M., Blaton N. (2001). Physical stabilisation of amorphous ketoconazole in solid dispersions with polyvinylpyrrolidone K25. Eur J Pharm Sci.

[7] Oksanen C.A., Zografi G. (1990). The relationship between the glass transition temperature and water vapor absorption by poly (vinylpyrrolidone). Pharm Res (N Y).

[8] Aso Y., Yoshioka S., Zhang J., Zografi G. (2002). Effect of water on the molecular mobility of sucrose and poly (vinylpyrrolidone) in a colyophilized formulation as measured by 13C-NMR relaxation time. Chem Pharm Bull.

[9] Taylor L.S., Zografi G. (1997). Spectroscopic characterization of interactions between PVP and indomethacin in amorphous molecular dispersions. Pharm Res (N Y).

[10] Miyazaki T., Yoshioka S., Aso Y., Kojima S. (2004). Ability of polyvinylpyrrolidone and polyacrylic acid to inhibit the crystallization of amorphous acetaminophen. J Pharm Sci.

[11] Aso Y., Yoshioka S. (2006). Molecular mobility of nifedipine− PVP and phenobarbital− PVP solid dispersions as measured by 13C-NMR spin-lattice relaxation time. J Pharm Sci.

[12] Marsac P.J., Konno H., Taylor L.S. (2006). A comparison of the physical stability of amorphous felodipine and nifedipine systems. Pharm Res (N Y).

[13] Konno H., Handa T., Alonzo D.E., Taylor L.S. (2008). Effect of polymer type on the dissolution profile of amorphous solid dispersions containing felodipine. Eur J Pharm Biopharm.

[14] Schenck L., Lowinger M., Troup G.M., Li L., McKelvey C. (2019). Achieving a Hot Melt Extrusion Design Space for the Production of Solid Solutions.

[15] Keratichewanun S., Yoshihashi Y., Sutanthavibul N., Terada K., Chatchawalsaisin J. (2015). An investigation of nifedipine miscibility in solid dispersions using Raman spectroscopy. Pharm Res (N Y).

[16] Caron V., Hu Y., Tajber L. (2013). Amorphous solid dispersions of sulfonamide/Soluplus® and sulfonamide/PVP prepared by ball milling. AAPS PharmSciTech.

[17] Trasi N.S., Bhujbal S.V., Zhou Q.T., Taylor L.S. (2019). Amorphous solid dispersion formation via solvent granulation–A case study with ritonavir and lopinavir. Int J Pharm X.

[18] Singh A., Van den Mooter G. (2016). Spray drying formulation of amorphous solid dispersions. Adv Drug Del Rev.

[19] Sarode A.L., Sandhu H., Shah N., Malick W., Zia H. (2013). Hot melt extrusion (HME) for amorphous solid dispersions: predictive tools for processing and impact of drug–polymer interactions on supersaturation. Eur J Pharm Sci.

[20] Sosnik A., Seremeta K.P. (2015). Advantages and challenges of the spray-drying technology for the production of pure drug particles and drug-loaded polymeric carriers. Adv Colloid Interface Sci.

[21] Sekikawa H., Fujiwara J., Naganuma T., Nakano M., Arita T. (1978). Dissolution behaviors and gastrointestinal absorption of phenytoin in phenytoin-polyvinylpyrrolidone coprecipitate. Chem Pharm Bull.

[22] Simonelli A., Mehta S., Higuchi W. (1969). Dissolution rates of high energy polyvinylpyrrolidone (PVP)-sulfathiazole coprecipitates. J Pharm Sci.

[23] Sertsou G., Butler J., Scott A., Hempenstall J., Rades T. (2002). Factors affecting incorporation of drug into solid solution with HPMCP during solvent change co-precipitation. Int J Pharm.

[24] Hu Q., Choi D.S., Chokshi H., Shah N., Sandhu H. (2013). Highly efficient miniaturized coprecipitation screening (MiCoS) for amorphous solid dispersion formulation development. Int J Pharm.

[25] Dong Z., Chatterji A., Sandhu H., Choi D.S., Chokshi H., Shah N. (2008). Evaluation of solid state properties of solid dispersions prepared by hot-melt extrusion and solvent co-precipitation. Int J Pharm.

[26] Sertsou G., Butler J., Hempenstall J., Rades T. (2002). Solvent change co-precipitation with hydroxypropyl methylcellulose phthalate to improve dissolution characteristics of a poorly water-soluble drug. J Pharm Pharmacol.

[27] Zhou H., Wang W., Hu H. (2019). Co-precipitation of calcium carbonate and curcumin in an ethanol medium as a novel approach for curcumin dissolution enhancement. J Drug Deliv Sci Technol.

[28] Shah N., Iyer R.M., Mair H.-J. (2013). Improved human bioavailability of vemurafenib, a practically insoluble drug, using an amorphous polymer-stabilized solid dispersion prepared by a solvent-controlled coprecipitation process. J Pharm Sci.

[29] Shah N., Sandhu H., Phuapradit W. (2012). Development of novel microprecipitated bulk powder (MBP) technology for manufacturing stable amorphous formulations of poorly soluble drugs. Int J Pharm.

[30] Rumondor A.C., Stanford L.A., Taylor L.S. (2009). Effects of polymer type and storage relative humidity on the kinetics of felodipine crystallization from amorphous solid dispersions. Pharm Res (N Y).

[31] Rumondor A.C., Wikström H., Van Eerdenbrugh B., Taylor L.S. (2011). Understanding the tendency of amorphous solid dispersions to undergo amorphous–amorphous phase separation in the presence of absorbed moisture. AAPS PharmSciTech.

[32] Patel K., Sarma V., Vavia P. (2013). Design and evaluation of Lumefantrine–Oleic acid self nanoemulsifying ionic complex for enhanced dissolution. J Pharm Sci.

[33] Mizuno Y., Kato Y., Kudo K., Kano S. (2009). First case of treatment failure of artemether-lumefantrine in a Japanese traveler with imported falciparum malaria. J Infect Dis.

[34] Repetto E.C., Traverso A., Giacomazzi C.G. (2011). Possible clinical failure of artemether-lumefantrine in an Italian traveler with uncomplicated falciparum malaria. Mediterr J Hematol Infect Dis.

[35] Jermain S.V., Lowinger M.B., Ellenberger D.J., Miller D.A., Su Y., Williams R.O. (2020). In vitro and in vivo behavior of KinetiSol® and spray dried amorphous solid dispersions of a weakly basic drug and ionic polymer. Mol Pharm.

[36] Sun D.D., Wen H., Taylor L.S. (2016). Non-sink dissolution conditions for predicting product quality and in vivo performance of supersaturating drug delivery systems. J Pharm Sci.

[37] Arun R., Smith A.A. (2011). Simultaneous HPLC-UV method for the estimation of artemether and lumefantrine in tablet dosage form. Int J Pharm Biomed Res.

[38] Song Y., Zemlyanov D., Chen X. (2016). Acid-base interactions in amorphous solid dispersions of lumefantrine prepared by spray-drying and hot-melt extrusion using X-ray photoelectron spectroscopy. Int J Pharm.

[39] Trasi N.S., Bhujbal S.V., Zemlyanov D.Y., Zhou Q.T., Taylor L.S. (2020). Physical stability and release properties of lumefantrine amorphous solid dispersion granules prepared by a simple solvent evaporation approach. Int J Pharm.

[40] Yoshioka M., Hancock B.C., Zografi G. (1994). Crystallization of indomethacin from the amorphous state below and above its glass transition temperature. J Pharm Sci.

[41] Pandi P., Bulusu R., Kommineni N., Khan W., Singh M. (2020). Amorphous solid dispersions: an update for preparation, characterization, mechanism on bioavailability, stability, regulatory considerations and marketed products. Int J Pharm.

[42] Singh G., Kaur L., Gupta G., Sharma S. (2017). Enhancement of the solubility of poorly water soluble drugs through solid dispersion: a comprehensive review. Indian J Pharm Sci.

[43] Hancock B.C., Zografi G. (1994). The relationship between the glass transition temperature and the water content of amorphous pharmaceutical solids. Pharm Res (N Y).

[44] Li N., Cape J.L., Mankani B.R. (2020). Water-induced phase separation of spray dried amorphous solid dispersions. Mol Pharm.

[45] Song Y., Yang X., Chen X., Nie H., Byrn S., Lubach J.W. (2015). Investigation of drug–excipient interactions in lapatinib amorphous solid dispersions using solid-state NMR spectroscopy. Mol Pharm.

[46] Qian F., Huang J., Hussain M.A. (2010). Drug–polymer solubility and miscibility: stability consideration and practical challenges in amorphous solid dispersion development. J Pharm Sci.

[47] Mann A.K., Schenck L., Koynov A. (2018). Producing amorphous solid dispersions via co-precipitation and spray drying: impact to physicochemical and biopharmaceutical properties. J Pharm Sci.

