# Supplementary material for: Physical Stability and Dissolution of Lumefantrine Amorphous Solid Dispersions Produced by Spray Anti-Solvent Precipitation
Source: J Pharm Sci. 2021 Jun;110(6):2423–31. doi: 10.1016/j.xphs.2020.12.033 (PMC8141512; doi:10.1016/j.xphs.2020.12.033)
Supplement: Multimedia component 1 [file mmc1.docx]

**SUPPLEMENTARY DATA**

**Figure S1.** PXRD plot showing the stability of amorphous lumefantrine (in the absence of polymers) under accelerated storage conditions. Crystallization can be seen from Day 1.

**
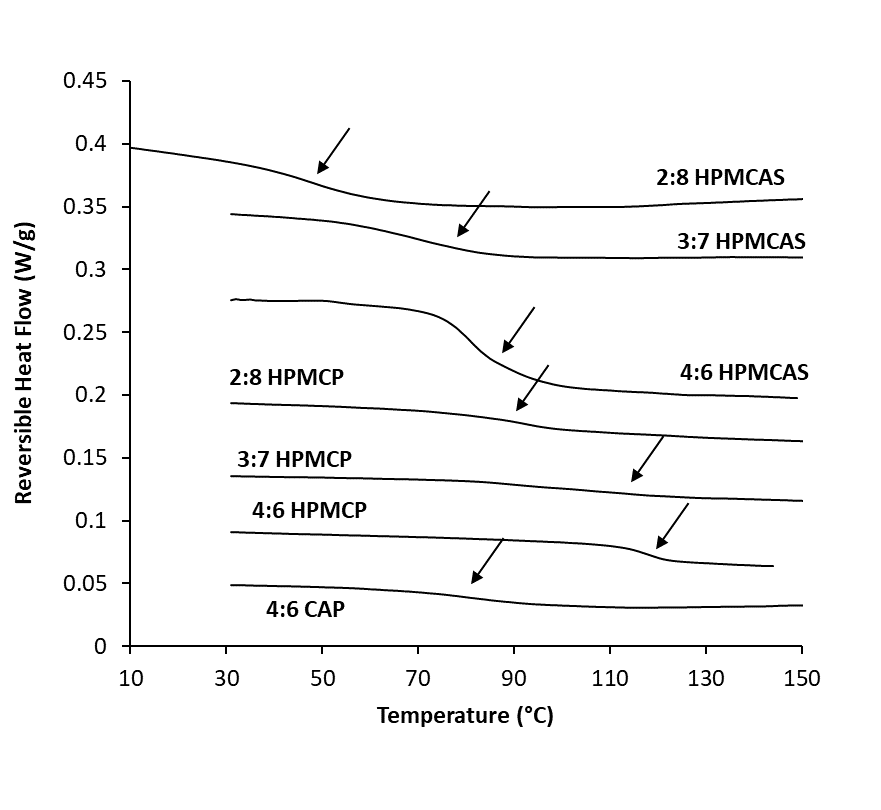
**

**Figure S2.** DSC curves of anti-solvent precipitated ASDs. The arrows indicate the glass transition temperature (T_g_).

**Table S1.** Flow characteristics of 2:8 Lum:HPMCP and 4:6 Lum:HPMCP dispersions (n=3).

| Sample |  | Bulk density (g/cm^3^) | Tapped density (g/cm^3^) | Carr’s Index | Hausner’s Ratio | Flow behavior |
| --- | --- | --- | --- | --- | --- | --- |
| 2:8 Lum-HPMCP |  | 0.53 ± 0.00 | 0.57 ± 0.01 | 7.1 ± 1.2 | 1.1 ± 0.0 | Excellent |
| 4:6 Lum-HPMCP |  | 0.52 ± 0.02 | 0.56 ± 0.01 | 6.3 ± 3.7 | 1.1 ± 0.0 | Excellent |
